# Overexpression of LIM and SH3 Protein 1 Leading to Accelerated G2/M Phase Transition Contributes to Enhanced Tumourigenesis in Oral Cancer

**DOI:** 10.1371/journal.pone.0083187

**Published:** 2013-12-26

**Authors:** Fumie Shimizu, Masashi Shiiba, Katsunori Ogawara, Ryota Kimura, Yasuyuki Minakawa, Takao Baba, Satoshi Yokota, Dai Nakashima, Morihiro Higo, Atsushi Kasamatsu, Yosuke Sakamoto, Hideki Tanzawa, Katsuhiro Uzawa

**Affiliations:** 1 Department of Clinical Molecular Biology, Graduate School of Medicine, Chiba University, Chiba, Japan; 2 Department of Clinical Oncology, Graduate School of Medicine, Chiba University, Chiba, Japan; 3 Division of Dentistry and Oral-Maxillofacial Surgery, Chiba University Hospital, Chiba, Japan; Deutsches Krebsforschungszentrum, Germany

## Abstract

**Background:**

LIM and SH3 protein 1 (LASP-1) is a specific focal adhesion protein involved in several malignant tumors. However, its role in oral squamous cell carcinoma (OSCC) is unknown. The aim of this study was to characterize the role and molecular status/mechanism of LASP-1 in OSCC.

**Methods:**

We evaluated *LASP-1* mRNA and protein expressions in OSCC-derived cell lines and primary OSCCs. Using an shRNA system, we analyzed the effect of LASP-1 on the biology and function of the OSCC cell lines, HSC-3 and Ca9-22. The cells also were subcutaneously injected to evaluate tumor growth *in vivo*. Data were analyzed by the Fisher’s exact test or the Mann-Whitney U test. Bonferroni correction was used for multiple testing.

**Results:**

Significant up-regulation of LASP-1 was detected in OSCC-derived cell lines (n = 7, *P*<0.007) and primary OSCCs (n = 50, *P*<0.001) compared to normal controls. LASP-1 knockdown cells significantly inhibited cellular proliferation compared with shMock-transfected cells (*P*<0.025) by arresting cell-cycle progression at the G2 phase. We observed dramatic reduction in the growth of shLASP-1 OSCC xenografts compared with shMock xenografts *in vivo*.

**Conclusion:**

Our results suggested that overexpression of LASP-1 is linked closely to oral tumourigenicity and further provide novel evidence that LASP-1 plays an essential role in tumor cellular growth by mediating G2/M transition.

## Introduction

Considerable evidence has suggested that sudden increases in cellular proliferation occur due to disruption of the cell-cycle control mechanism. Previous studies have reported a correlation between cell-cycle regulation and development of oral squamous cell carcinomas (OSCCs) [1], [2], [3]. Despite accumulating data, the precise mechanism of the clinical benefit of cell-cycle regulation in OSCCs has not been elucidated.

Cancer cell proliferation happens as a result of the disruption of the cell-cycle control mechanisms and continuous signaling for mitosis. Recent studies have reported that dysregulation in G2/M transition promotes cellular proliferation in many tumor types [4], [5], [6]. Among the genes correlated with the G2/M phase, nucleic LIM and SH3 protein (LASP-1) at the G2/M phase is considered essential for oncogenic activity in patients with breast cancer [7]. LASP-1 was identified originally from a cDNA library of metastatic breast cancer metastases [8]. It is localized within multiple sites of dynamic F-actin assembly such as focal contacts [9] and is involved in cytoskeletal architecture [10]. In human cancer derived cells, while silencing of LASP-1 resulted in a strong inhibition of cellular growth [11], overexpression of LASP-1 significantly promoted tumor growth *in vivo* [12]. Moreover, overexpression of LASP-1 has been observed in a variety of malignant tumors such as metastatic breast cancer [11], ovarian cancer [13], bladder cancer [14], medulloblastoma [15], and hepatocellular carcinoma [16].

Recent studies have demonstrated LASP-1 together with another focal adhesion protein plays an important role in G2/M transition by inhibiting cdc2 [17], [18], suggesting its possible relation to cell-cycle in cancer. Based on these observations we hypothesized that LASP-1 is closely related to oral tumorigenesis with accelerated G2/M transition.

In the current study, we found aberrant expression of LASP-1 and evaluated the correlation between its expression and clinicopathological characteristics in OSCCs. We also performed functional analysis to define the biologic effects of LASP-1.

## Materials and Methods

### Ethics Statement

The study protocol was approved by the Ethical Committee of Graduate School of Medicine, Chiba University (The approval number, 236) and was performed in accordance with the ethical standards laid down in the Declaration of Helsinki. Written informed consent was received from all patients.

All experimental animals were treated and cared for in accordance with the guidelines of Chiba University. Experimental animals were sacrificed by cervical dislocation. We made every effort to relieve the pain of experimental animals. The protocol was approved by the Committee on the Ethics of Animal Experiments of Chiba University (The approval number, 25–221).

### Cell Culture and Tissue Samples

The human OSCC cell lines (HSC-3, KON, and HO-1-N-1) were purchased from the Human Science Research Resources Bank, Osaka, Japan. RIKEN BRC (Tsukuba, Japan) provided Sa3, HO-1-u-1, HSC-4, and Ca9-22 through the National Bio-Resource Project of the Ministry of Education, Culture, Sports, Science and Technology in Japan. Cellular identity was confirmed by short tandem repeat profiling. Primary cultured human normal oral keratinocytes (HNOKs) were obtained from three healthy donors and served as the normal controls [19], [20], [21]. All cells were cultured in Dulbecco’s modified Eagle’s medium (Sigma, St. Louis, MO, USA) supplemented with 10% fetal bovine serum (Sigma) and 50 units/ml penicillin and streptomycin (Sigma) in a humidified atmosphere of 5% carbon dioxide/air at 37°C.

Fifty pairs of primary OSCC samples and corresponding normal oral epithelial tissues were obtained intraoperatively at Chiba University Hospital. The institutional review board of Chiba University approved this study. Informed consent was obtained from all the patients. The resected tissues were divided for RNA isolation and immunohistochemistry (IHC); the former was frozen immediately and stored at –80°C, the latter was fixed in 20% buffered formaldehyde solution. Each tissue was diagnosed histopathologically according to the World Health Organization criteria by the Department of Pathology, Chiba University Hospital. Clinicopathological staging was determined according to the tumor-node-metastases classification of the International Union against Cancer. All OSCC samples were confirmed histologically that tumor was present in over 90% of the specimens.

### mRNA Expression Analysis

Total RNA was extracted using Trizol Reagent (Invitrogen, Carlsbad, CA, USA) according to the manufacturer’s instructions. cDNA was generated from 5 µg of total RNA using Ready-To-Go You-Prime First-Strand Beads (GE Healthcare, Buckinghamshire, UK) and oligo (dT) primer (Hokkaido System Science, Sapporo, Japan). Real-time quantitative reverse transcriptase polymerase chain reaction (qRT-PCR) was performed using LightCycler® 480 PCR system (Roche Diagnostics GmbH, Mannheim, Germany) to evaluate the expression level of *LASP-1* mRNA in the OSCC-derived cell lines and HNOKs. The primer sequences used for qRT-PCR were: *LASP-1*, forward, 5′-CAGCCCCAGTCTCCATACAG-3′; reverse, 5′-ATACTGATGTCGCGGCGG-3′; and universal probe #77. Primers and probes were designed using the ProbeFinder qPCR Assay Design Software, available at www.universalprobelibrary.com. The qRT-PCR amplifications were carried out as follows: initial denaturation at 95°C for 10 minutes, 45 cycles of amplification at 95°C for 10 seconds, denaturation at 55°C for 30 seconds for annealing/extension, and cooling at 40°C for 30 seconds. The transcript amount for the *LASP-1* was calculated from the respective standard curves and normalized to the *glyceraldehyde-3-phosphate dehydrogenase* (*GAPDH*) (forward 5′-CATCTCTGCCCCCTCTGCTGA-3′cell, reverse 5′-GGATGACCTTGCCCACAGCCT-3′, and universal probe #60) transcript amount determined in corresponding samples.

### Immunoblotting Analysis

The cells were lysed in buffer (7 M urea, 2 M thiourea, 4% w/v CHAPS, and 10 mM Tris [pH 7.4]) with the proteinase inhibitor cocktail (Roche) and incubated at 4°C for 30 minutes. The protein concentration was evaluated using a Bio-Rad Protein Assay (Bio-Rad Laboratories, Hercules, CA, USA). Protein extracts were separated by sodium dodecyl sulfate polyacrylamide gel electrophoresis in 10% gel and transferred to nitrocellulose membranes before incubation overnight with primary antibodies against LASP-1 (Applied Biological Materials, Richmond, BC, Canada), cyclin A, cyclin B, or phospho-cdc2 (Tyr15) (Cell Signaling Technology, Danvers, MA) at 4°C. The membrane was washed with 0.1% Tween-20 in Tris-buffered saline, incubated with secondary antibodies, horseradish peroxidase-conjugated anti-rabbit or anti-mouse IgG (Promega, Madison, WI, USA) for 1 hour at room temperature. SuperSignal Chemiluminescent substrate (Thermo, Waltham, MA, USA) was used for protein detection. The immunoreactive bands were visualized on a cooled CCD camera system (ATTO, Tokyo, Japan), and the CS Analyzer software version 3.0 (ATTO) was used for signal intensity quantitation.

### IHC

IHC was performed on paraffin-embedded specimens cut into 4-µm sections using rabbit anti-LASP-1 polyclonal antibody (Applied Biological Materials). The endogenous peroxidase activity was blocked by a 30-minute incubation in 0.3% hydrogen peroxide solution in 100% methanol. The sections were incubated in 1.5% blocking serum (Santa Cruz Biotechnology, Inc., Dallas, TX, USA) in phosphate-buffered saline (PBS) for 2 hours at room temperature before reacting overnight with anti-LASP-1 antibody (1∶1000 dilution) at 4°C in a moist chamber. Before incubation with the primary antibody, the slides were washed three times in PBS and treated with Histofine Simplestain Max-PO (G) (Nichirei, Tokyo, Japan). 3,3′-Diaminobenzidine tetrahydrochloride (DAKO, Carpinteria, CA, USA) was used as a chromogen, and the tissue sections were counterstained lightly with hematoxylin, dehydrated with ethanol, cleaned with xylene, and mounted with Marinol (Muto Pure Chemicals, Tokyo, Japan). To avoid nonspecific binding of an antibody to proteins other than antigen, an immunizing peptide blocking experiment was performed. Triplicate sections were immunostained without reacting primary antibody as a negative control to confirm staining specificity.

To determine LASP-1 expression levels, the slides were evaluated using a IHC scoring systems described previously [22], [23]. The staining intensities were scored as 1, weak; 2, moderate; and 3, strong. The IHC score was calculated by multiplying the percentage of the positive tumor cells and the staining intensity. Cases with a score exceeding 113.0 (+3 standard deviation [SD] score for normal tissue) were defined as LASP-1-positive. The ±3 SD cutoff, which statistically is just 0.2% of the measurement and is expected to fall outside this range, was used because it was unlikely to be affected by a random experimental error produced by sample manipulation [24]. Two independent pathologists who had no knowledge of the patients’ clinical status made these judgments.

### Transfection with shRNA Plasmid

The OSCC cell lines, HSC-3 and Ca9-22, with higher LASP-1 protein expression were stably transfected with the LASP-1 shRNA (shLASP-1) or the control shRNA (shMock) (Santa Cruz Biotechnology, Inc.) using Lipofectamine LTX and Plus Reagents (Invitrogen). Transfected cells were selected in medium containing 1 µg/ml Puromycin (Invitrogen).

### Cellular Proliferation Assays

To determine the effect LASP-1 knockdown on cellular proliferation, shLASP-1- and shMock-transfected cells were seeded in six-well plates at 1×10^4^ cells/well, trypsinized, and counted in triplicate every day for 7 days using a hemocytometer.

### Cell-cycle Analysis

Seven days after the establishment of LASP-1 knockdown cells, cell-cycle distribution was checked. To synchronize cells at the G2/M transition, the cells were treated with 200 ng/ml nocodazole (Sigma) for 20 hours [25]. The cells were pelleted, rinsed with PBS, and probed with CycleTEST Plus DNA reagent kit (Becton-Dickinson, San Jose, CA, USA), according to the manufacturer’s protocol. After centrifugation at 400×g for 5 minutes, 250 microliters of solution A (trypsin buffer) was added, and the mixture was incubated for 10 minutes at room temperature. Two hundred microliters of solution B (trypsin inhibitor and RNase in a buffer) then was added and the mixture was incubated for 10 minutes at room temperature. Finally, 200 microliters of solution C (propidium iodide stain solution) was added, and the mixture was incubated in the dark for 10 minutes on ice. Flow cytometric determination of DNA content was analyzed using the Accuri C6 Flow Cytometer (Becton-Dickinson). Cell-cycle distributions were quantified using FCS Express 4 (De Novo Software, Los Angeles, CA, USA). The experiments were performed in triplicates.

### *In vivo* Tumor Growth Assay

To evaluate cancer growth *in vivo*, 2×10^7^ shLASP-1- and shMock-transfected cells were independently injected subcutaneously into the backs of female nude mice, BALB/c-nu, purchased from Oriental Yeast Co. (Tokyo, Japan). All experimental animals were treated and cared for in accordance with institutional guidelines. The tumors were measured using calipers every 3 to 4 days after they reached a volume of 100 mm^3^. The tumor volume was calculated using the formula 4π/3×(width/2)^2^×(length/2). The mice were sacrificed after 28 days. Tumor tissues were fixed in 10% formalin, and paraffin sections (4 µm) were prepared for immunohistochemistry of LASP-1 and hematoxylin and eosin (H&E) staining.

### Statistical Analysis

Statistical significance was determined using Fisher’s exact test or the Mann-Whitney U test. *P*<0.05 was considered significant. Bonferroni correction was utilized for multiple testing. The data are expressed as the mean ± standard error of the mean (SEM).

## Results

### Evaluation of LASP-1 Expression in OSCC-derived Cell Lines

**Figure 1:**
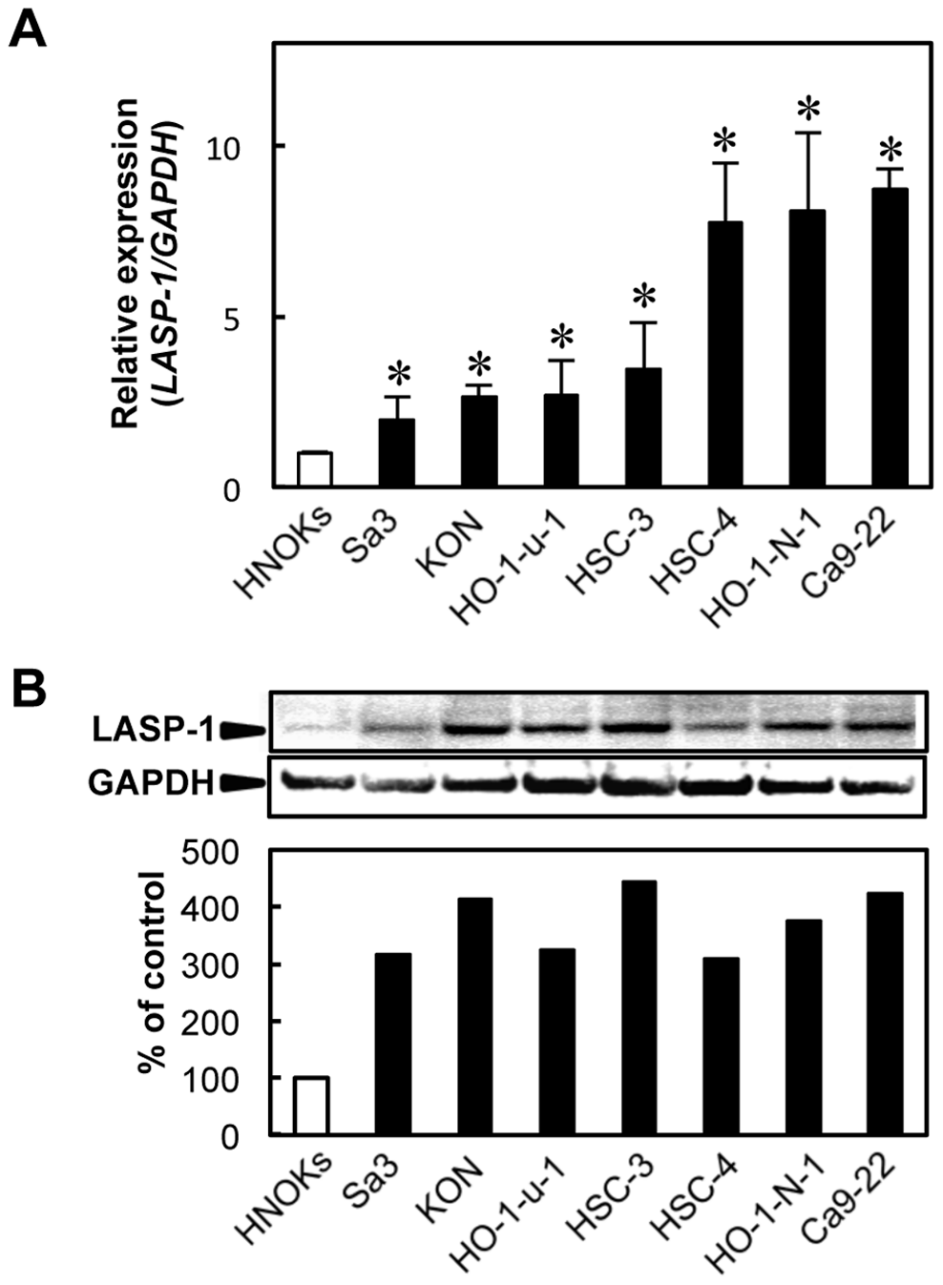
Evaluation of LASP-1 expression in OSCC-derived cell lines. (A) Quantification of *LASP-1* mRNA expression in OSCC-derived cell lines by qRT-PCR analysis. mRNA expression levels are normalized to GAPDH. Significant up-regulation of *LASP-1* mRNA is seen in seven OSCC-derived cell lines compared with that in HNOKs. Data are expressed as the means ± SEM of triplicate results (*P<0.007; Mann-Whitney U test with Bonferroni correction). (B) Immunoblotting analysis of LASP-1 protein in the OSCC-derived cell lines and HNOKs. LASP-1 protein expression is up-regulated in the OSCC-derived cell lines compared with that in the HNOKs. Densitometric LASP-1 protein data are normalized to GAPDH protein levels. The values are expressed as a percentage of the HNOKs.

To analyze the expression status of *LASP-1*, we performed qRT-PCR and immunoblotting analyses using seven OSCC-derived cell lines (HSC-3, HSC-4, KON, HO-1-u-1, HO-1-N-1, Ca9-22, and Sa3) and HNOKs. *LASP-1* mRNA was significantly (*P*<0.007) up-regulated in all OSCC cell lines compared with that in the HNOKs (Figure 1A). Figure 1B shows representative results of immunoblotting analyses. A significant increase in LASP-1 protein expression was seen in all OSCC-derived cell lines compared with the HNOKs. Expression analysis indicated that both transcription and translation products of LASP-1 were highly expressed in OSCC-derived cell lines.

### Evaluation of LASP-1 Expression in Primary OSCCs

**Figure 2:**
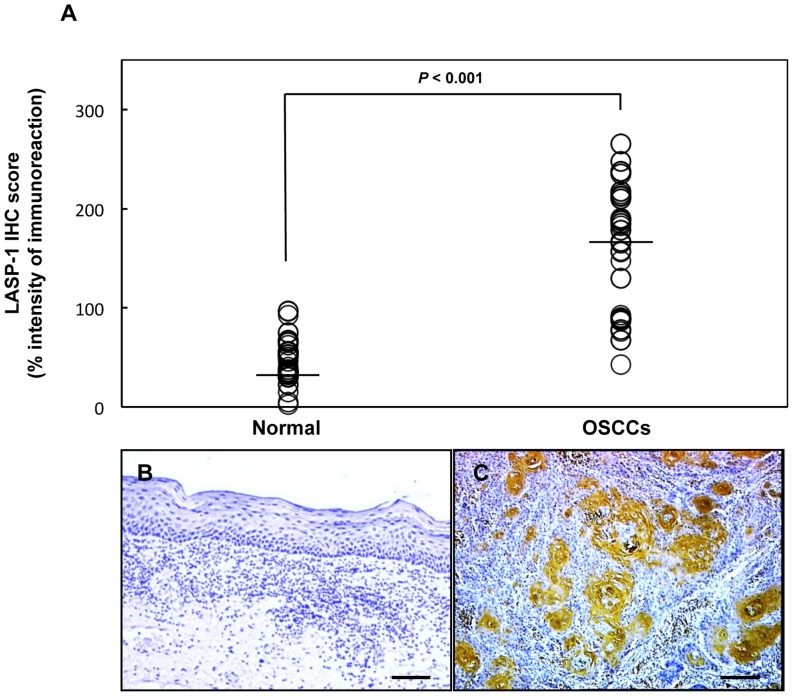
Evaluation of LASP-1 protein expression in primary OSCCs. (A) The status of LASP-1 protein expression in primary OSCCs (n = 50) and the normal counterparts based on an IHC scoring system. IHC scores are calculated as follows: IHC score = 1×(number of weakly stained cells in the field)+2×(number of moderately stained cells in the field)+3×(number of intensely stained cells in the field). The LASP-1 IHC scores for normal oral tissues and OSCCs range from 2.5 to 97 (median, 35) and 43 to 265 (median, 178), respectively. LASP-1 protein expression levels in OSCCs are significantly higher than in normal oral tissues (*P<0.001; Mann-Whitney U test). (B, C) Representative IHC results of LASP-1 in normal oral tissues and primary OSCCs. Original magnification, ×100. Scale bars, 5 µm. LASP-1 is highly overexpressed in OSCCs compared to normal oral tissues.

To find out the expression status of LASP-1 in primary OSCCs and its relations to the clinicopathological characteristics, we investigated LASP-1 protein expression in primary OSCCs and paired normal oral tissues from 50 patients using the IHC scoring system. Strong LASP-1 immunoreactivity was detected in the cytoplasm of the OSCCs, whereas the normal tissues showed negative immunostaining (Figure 2B, C). The LASP-1 IHC scores ranged from 43 to 265 in OSCCs (median, 178) and 2.5 to 97 in normal oral tissues (median, 35). IHC scores in primary OSCCs were significantly (*P*<0.05) higher than those in the normal oral tissues (Figure 2A).

Table 1 shows the correlations between the clinicopathological characteristics of the patients with OSCC and the status of LASP-1 protein expression using the IHC scoring system. Among the clinical classifications, the LASP-1 positive OSCCs were correlated significantly (*P = *0.02) with tumor size. Furthermore, a significant (*P = *0.04) difference was found in the LASP-1 expression levels between the T1/T2 group and the T3/T4 group, indicating LASP-1 expression levels are higher in advanced stages compared with early stages. No significant relations were found in age, gender, N-regional lymph node, histopathological type, and tumor site.

**Table 1:** Correlations between clinicopathological characteristics of patients with OSCC and LASP-1 protein expression levels.

		Results of immunostaining			
		No. of patients (%)			
Parameter	Total	LASP1 (−)		LASP1 (+)	p-value
Age at surgery					
<60	12	3 (25)		9 (75)	0.66
60∼70	19	8 (42)		11 (58)	
70>	19	7 (37)		12 (63)	
Gender					
Male	22	8 (36)		14 (64)	0.96
Female	28	10 (36)		18 (64)	
T-primary tumor size					
T1	9	5 (56)		4 (44)	*0.02
T2	14	7 (50)		7 (50)	
T3	16	5 (31)		11 (69)	
T4	11	1 (9)		10 (91)	
T1+T2	23	12 (52)		11 (48)	*0.04
T3+T4	27	6 (28)		21 (72)	
N-regional lymph node					
N(−)	18	7 (39)		11 (61)	0.77
N(+)	32	11 (34)		21 (66)	
Stage					
I	8	4 (50)		4 (50)	0.10
II	10	3 (30)		7 (70)	
III	19	10 (53)		9 (47)	
IV	13	1 (8)		12 (92)	
I+II	18	7 (39)		11 (61)	0.77
III+IV	32	11 (34)		21 (66)	
Histopathological type					
Well differentiated	24	9 (38)	15 (62)		0.94
Moderatelydifferentiated	18	6 (33)	12 (67)		
Poorly differentiated	8	3 (38)	5 (62)		
Tumor site					
Tongue	24	8 (33)	16 (67)		0.66
Gingiva	19	7 (37)	12 (63)		
Buccal mucosa	3	1 (33)	2 (67)		
Soft palate	2	1 (50)	1 (50)		
Oral floor	2	1 (50)	1 (50)		
Total	50	18 (36)	32 (64)		

P<0.05.

### Establishment of LASP-1 Knockdown Cells

**Figure 3:**
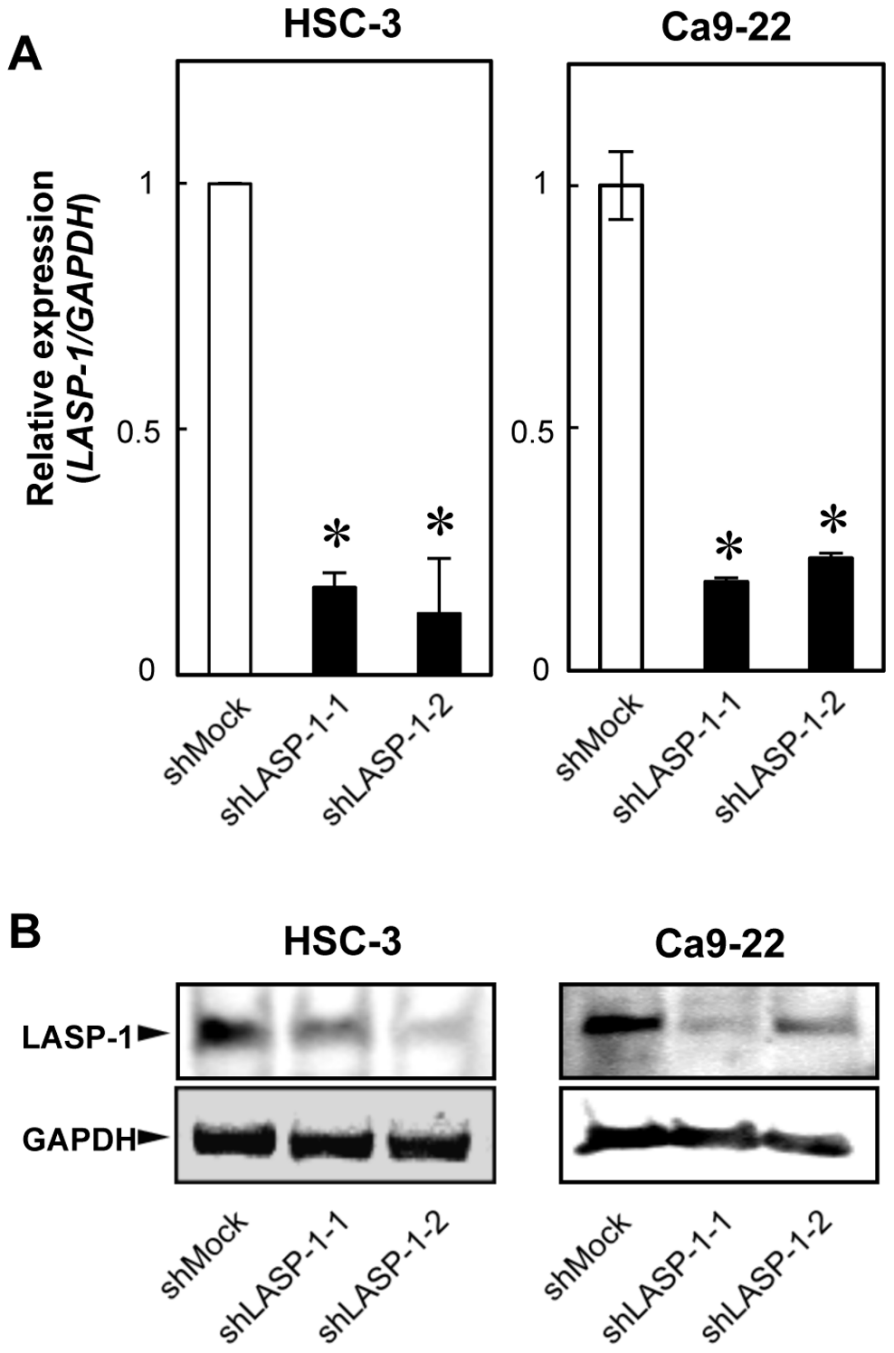
Expression of LASP-1 in shLASP-1-transfected cells. (A) Expression of *LASP-1 *mRNA in shMock- and shLASP-1-transfected cells (HSC-3- and Ca9-22-derived transfectants; 2 clones each). The *LASP-1* mRNA expression in the shLASP-1 is significantly (*P<0.025; Mann-Whitney U test with Bonferroni correction) lower than that in the shMock-transfected cells. (B) Immunoblotting analysis of LASP-1 protein in the shMock- and shLASP-1-transfected cells (HSC-3- and Ca9-22-derived transfectants; 2 clones each). The LASP-1 protein in the shLASP-1 transfected cells is depleted markedly compared with the shMock-transfected cells.

To study the possible function of LASP-1 in OSCCs, the OSCC-derived cell lines, HSC-3 and Ca9-22, were transfected with LASP-1 shRNA and the control shRNA (shMock). The expression levels of *LASP-1* mRNA and protein in shLASP-1-transfected cells showed significant (*P*<0.025) decrease compared with the shMock-transfected cells (Figure 3A, B).

### Reduced Cellular Growth in LASP-1 Knockdown Cells

**Figure 4:**
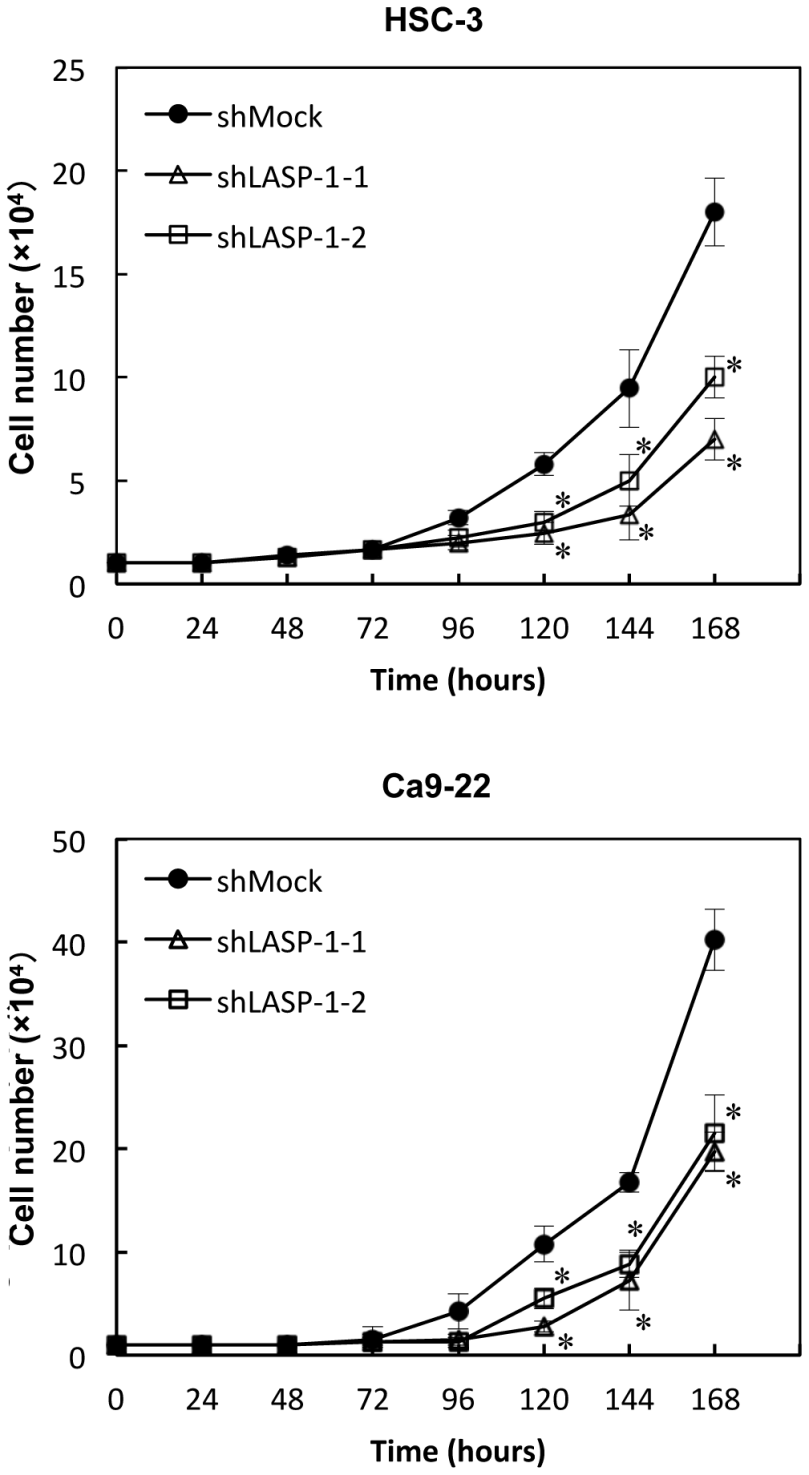
Proliferation of shLASP-1-transfected cells. To determine the effect of shLASP-1 on cellular proliferation, shLASP-1- and shMock-transfected cells were seeded in six-well plates at a density of 1×10^4^ viable cells/well. The shLASP-1 transfected HSC-3 and Ca9-22 cells have a significant (*P<0.025; Mann-Whitney U test with Bonferroni correction) decrease in cellular growth compared with the shMock-transfected cells.

To study the impact of LASP-1 on cellular proliferative ability, we monitored the cellular growth in shLASP-1-transfected cells for seven consecutive days. Both HSC-3 and Ca9-22 shLASP-1-transfected cells had a significant (*P*<0.025) decrease in cellular growth compared with the shMock-transfected cells (Figure 4).

### Knockdown of LASP-1 Induces G2 Phase Accumulation

**Figure 5:**
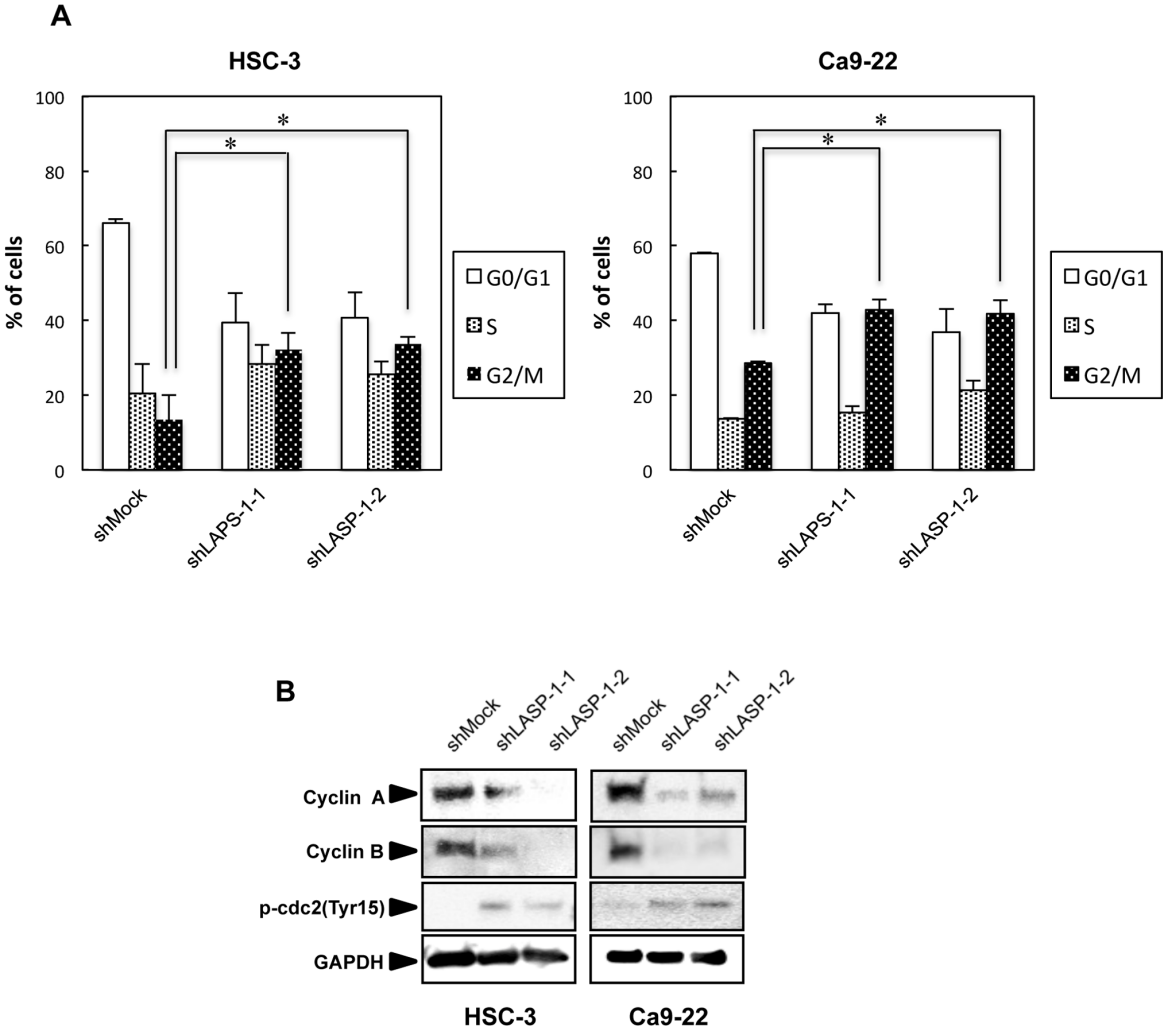
shLASP-1 promotes G2 arrest. (A) Flow cytometric determination of DNA content was analyzed using the Accuri C6 Flow Cytometer. shLASP-1-transfected HSC-3 and Ca9-22 cells show significant (*P<0.025; Mann-Whitney U test with Bonferroni correction) G2 phase accumulation as opposed to the shMock-transfected cells. (B) Immunoblotting analysis shows down-regulation of cyclin A and cyclin B and up-regulation of phospho-cdc2 in the knockdown cells.

To investigate the mechanism by which down-regulated LASP-1 is related to cell-cycle progression, we then investigated the cell-cycle distributions of shLASP-1-transfected cells using fluorescence-activated cell sorting (FACS). The proportion of cells in the G2 phase in the shLASP-1-transfected cells was significantly (*P*<0.025) higher than that in shMock-transfected cells (Figure 5A). Similar results were obtained in three independent experiments (Figure S1, S2), suggesting that down-regulation of LASP-1 inhibited cellular proliferation, which induced G2 arrest.

To identify the mechanism by which down-regulated LASP-1 blocks G2/M transition, we assessed the protein expression level of cyclin A, cyclin B, or phospho-cdc2 (Tyr15). The protein expressions of cyclin A and cyclin B were down-regulated while phospho-cdc2 (Tyr 15) was up-regulated (Figure 5B), indicating the arrest of the G2/M phase in the shLASP-1 transfected cells.

### LASP-1 Promoted Tumor Growth *in vivo*

**Figure 6:**
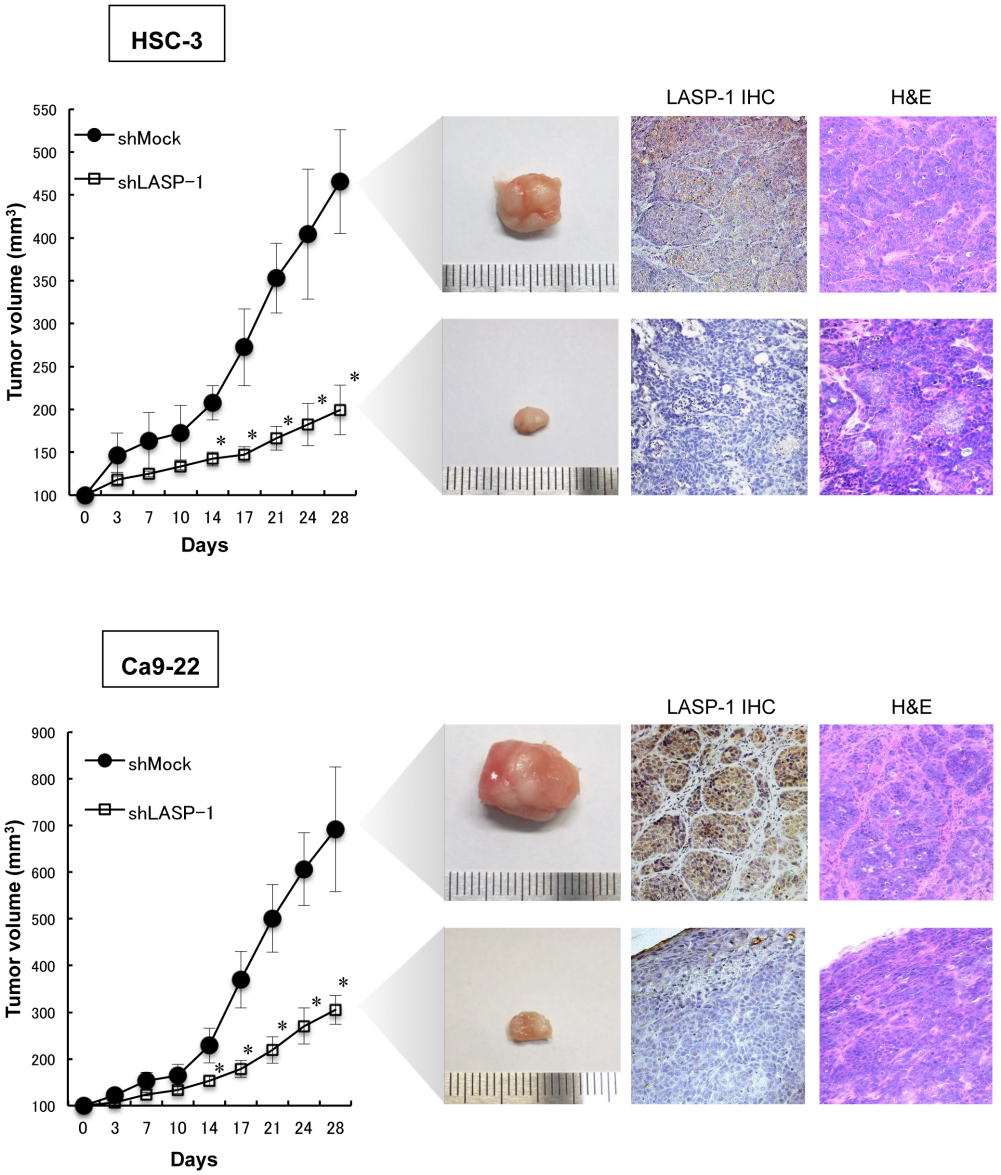
LASP-1 promotes tumor growth *in vivo*. shLASP-1- and shMock-transfected cells (HSC-3 and Ca9-22) were injected subcutaneously into the backs of female nude mice (n = 3). The tumor growth of the shLASP-1-injected mice is significantly (*P<0.05; Mann-Whitney U test) inhibited compared to the shMock-injected mice. Immunohistochemistry clearly demonstrated decreased immunostaining for LASP-1 in the shLASP-1-derived tumors than shMock injected mice. H&E staining confirmed the presence of tumor cells. Original magnification, ×200.

To define the effect of LASP-1 on cancer growth *in vivo*, shLASP-1- and shMock-transfected cells of the HSC-3 and Ca9-22 cell lines were injected subcutaneously into the backs of female nude mice, respectively (3 mice in each group). Consistent with our *in vitro* findings, the mean tumor volume of the shLASP-1 cells was significantly (*P*<0.05) smaller compared to the shMock-transfected cells. H&E staining confirmed the presence of tumor cells in all groups and LASP-1 immunohistochemistry of tumor sections demonstrated LASP-1 knockdown has been maintained *in vivo* (Figure 6). These results indicated that LASP-1 promotes tumor growth in nude mice.

## Discussion

In the current study, we found a high prevalence of increased LASP-1 expression in patients with OSCC and a significant correlation with the primary tumor size (Table 1). Furthermore, knockdown of LASP-1 in OSCC-derived cell lines resulted in a dramatic effect on growth inhibition *in vitro* and *in vivo* via arrest of the G2/M phase. The current data provided a novel insight into the role of aberrant LASP-1 function as an oncogenic process in this disease.

A significant up-regulation of LASP-1 was detected in all OSCC-derived cells examined at protein levels (Figure 1B), indicating that LASP-1 expression is necessary for oral carcinogenesis. On the other hand, the mRNA expression states depended on the cells (Figure 1A). A possible explanation for this discrepancy is that LASP-1 proteins are differently affected by the post-translational ubiquitination and proteolysis in each tumor cell lines. In this context, discrepancies between *LASP-1 *mRNA expression levels and protein levels has been reported in bladder cancer cell lines [14].

FACS analysis showed that LASP-1 is specifically active in the G2/M phase in OSCC cells (Figure 5A). The G2/M checkpoint is necessary for the cell to repair DNA damage before mitotic entry. When DNA is damaged, cdc2, an important regulator of cell progression, is inactivated through increasing phosphorylation residues of Tyr 15, causing the cells to arrest in the G2 phase [26]. Cyclin A and cyclin B are other crucial regulators of cell-cycle progression. Cyclin A associates with cdk2 and cdc2 and is required at the initiation of the S phase and the G2/M transition [27], [28]. Meanwhile, cyclin B associates with cdc2 and regulates mitotic entry and exit [29]. Expression levels of cyclin A and cyclin B are often up-regulated in a variety of tumors [30], [31]. Depletion of cyclin A and cyclin B leads to cell-cycle arrest in G2 [32], [33]. During the G2 phase, the cyclin B/cdc2 complex is inactivated by phosphorylation on Try15 and Thr14 of cdc2 by the Wee 1 and Myt 1 kinases [34], [35]. At the onset of mitosis, cdc25C phosphatase activates cdc2 by removing phosphates at Thr 14 and Try 15 [36], [37] and prevents the cyclin B1/cdc2 complex from being inactivated again [38].

Based on these observations, we next determined the expression status of the related genes mentioned previously. As expected, while cyclin A and B were significantly down-regulated, we detected up-regulation of phoshpo-cdc2 in the LASP-1 knockdown cells. This was consistent with previous studies that implied LASP-1 is responsible for cellular proliferation due to cell-cycle arrest at the G2 phase in patients with breast and ovarian cancers [11], [13].

In conclusion, our data indicated that LASP-1 may be associated with tumor progression by promoting cell-cycle progression in the G2 phase. It is noteworthy that our *in vivo* data showed dramatic inhibition of tumor growth by LASP-1 silencing, suggesting that this molecule may be a useful biomarker of proliferation and a possible therapeutic target for developing anti-cancer drugs in human OSCCs because most of our patients with an OSCC have overexpressed LASP-1 protein (Table 1).

## Supporting Information

Figure S1 **FACS analysis of shMock- and shLASP-1-transfected HSC-3 cells.** The percentage of the G2/M phase in shLASP-1-transfected HSC-3 cells was higher than in shMock-transfected cells.
(TIF)

Figure S2 **FACS analysis of shMock- and shLASP-1-transfected Ca9-22 cells.** The percentage of the G2/M phase in shLASP-1-transfected Ca9-22 cells was higher than in shMock-transfected cells.
(TIF)

## References

[1] Kudo Y, Takata T, Yasui W, Ogawa I, Miyauchi M, et al (1998) Reduced expression of cyclin-dependent kinase inhibitor p27Kip1 is an indicator of malignant behavior in oral squamous cell carcinoma. Cancer 83: 2447–2455. 9874448 10.1002/(sici)1097-0142(19981215)83:12<2447::aid-cncr7>3.0.co;2-a

[2] Hsu LC, Huang X, Seasholtz S, Potter DM, Gollin SM (2006) Gene amplification and overexpression of protein phosphatase 1alpha in oral squamous cell carcinoma cell lines. Oncogene 25: 5517–5526. 16619035 10.1038/sj.onc.1209563

[3] Shao Y, Qu Y, Dang S, Yao B, Ji M (2013) MiR-145 inhibits oral squamous cell carcinoma (OSCC) cell growth by targeting c-Myc and Cdk6. Cancer Cell Int 13: 51. 23710609 10.1186/1475-2867-13-51 PMC3680295

[4] Shi Y, Ding X, He ZH, Zhou KC, Wang Q, et al (2009) Critical role of TRPC6 channels in G2 phase transition and the development of human oesophageal cancer. Gut 58: 1443–1450. 19651628 10.1136/gut.2009.181735

[5] Schultz J, Ibrahim SM, Vera J, Kunz M (2009) 14-3-3sigma gene silencing during melanoma progression and its role in cell cycle control and cellular senescence. Mol Cancer 8: 53. 19642975 10.1186/1476-4598-8-53 PMC2723074

[6] Ignatov T, Modl S, Thulig M, Weißenborn C, Treeck O, et al (2013) GPER-1 acts as a tumor suppressor in ovarian cancer. J Ovarian Res 6: 51. 23849542 10.1186/1757-2215-6-51 PMC3723961

[7] Frietsch JJ, Grunewald TG, Jasper S, Kammerer U, Herterich S, et al (2010) Nuclear localisation of LASP-1 correlates with poor long-term survival in female breast cancer. Br J Cancer 102: 1645–1653. 20461080 10.1038/sj.bjc.6605685 PMC2883150

[8] Tomasetto C, Moog-Lutz C, Régnier CH, Schreiber V, Basset P, et al (1995) Lasp-1 (MLN 50) defines a new LIM protein subfamily characterized by the association of LIM and SH3 domains. FEBS Lett 373: 245–249. 7589475 10.1016/0014-5793(95)01040-l

[9] Lin YH, Park ZY, Lin D, Brahmbhatt AA, Rio MC, et al (2004) Regulation of cell migration and survival by focal adhesion targeting of Lasp-1. J Cell Biol 165: 421–432. 15138294 10.1083/jcb.200311045 PMC2172195

[10] Chew CS, Parente JA, Zhou C, Baranco E, Chen X (1998) Lasp-1 is a regulated phosphoprotein within the cAMP signaling pathway in the gastric parietal cell. Am J Physiol 275: C56–67. 9688835 10.1152/ajpcell.1998.275.1.C56

[11] Grunewald TG, Kammerer U, Schulze E, Schindler D, Honig A, et al (2006) Silencing of LASP-1 influences zyxin localization, inhibits proliferation and reduces migration in breast cancer cells. Exp Cell Res 312: 974–982. 16430883 10.1016/j.yexcr.2005.12.016

[12] Zhao L, Wang H, Liu C, Liu Y, Wang X, et al (2010) Promotion of colorectal cancer growth and metastasis by the LIM and SH3 domain protein 1. Gut 59: 1226–1235. 20660701 10.1136/gut.2009.202739

[13] Grunewald TG, Kammerer U, Winkler C, Schindler D, Sickmann A, et al (2007) Overexpression of LASP-1 mediates migration and proliferation of human ovarian cancer cells and influences zyxin localisation. Br J Cancer 96: 296–305. 17211471 10.1038/sj.bjc.6603545 PMC2359999

[14] Chiyomaru T, Enokida H, Kawakami K, Tatarano S, Uchida Y, et al (2012) Functional role of LASP1 in cell viability and its regulation by microRNAs in bladder cancer. Urol Oncol 30: 434–443. 20843712 10.1016/j.urolonc.2010.05.008

[15] Traenka C, Remke M, Korshunov A, Bender S, Hielscher T, et al (2010) Role of LIM and SH3 protein 1 (LASP1) in the metastatic dissemination of medulloblastoma. Cancer Res 70: 8003–8014. 20924110 10.1158/0008-5472.CAN-10-0592

[16] Wang H, Li W, Jin X, Cui S, Zhao L (2013) LIM and SH3 protein 1, a promoter of cell proliferation and migration, is a novel independent prognostic indicator in hepatocellular carcinoma. Eur J Cancer 49: 974–983. 23084841 10.1016/j.ejca.2012.09.032

[17] Hirota T, Morisaki T, Nishiyama Y, Marumoto T, Tada K, et al (2000) Zyxin, a regulator of actin filament assembly, targets the mitotic apparatus by interacting with h-warts/LATS1 tumor suppressor. J Cell Biol 149: 1073–1086. 10831611 10.1083/jcb.149.5.1073 PMC2174824

[18] Nix DA, Fradelizi J, Bockholt S, Menichi B, Louvard D, et al (2001) Targeting of zyxin to sites of actin membrane interaction and to the nucleus. J Biol Chem 276: 34759–34767. 11395501 10.1074/jbc.M102820200

[19] Kasamatsu A, Uzawa K, Nakashima D, Koike H, Shiiba M, et al (2005) Galectin-9 as a regulator of cellular adhesion in human oral squamous cell carcinoma cell lines. Int J Mol Med 16: 269–273. 16012760

[20] Endo Y, Uzawa K, Mochida Y, Shiiba M, Bukawa H, et al (2004) Sarcoendoplasmic reticulum Ca(2+) ATPase type 2 downregulated in human oral squamous cell carcinoma. Int J Cancer 110: 225–231. 15069686 10.1002/ijc.20118

[21] Shimada K, Uzawa K, Kato M, Endo Y, Shiiba M, et al (2005) Aberrant expression of RAB1A in human tongue cancer. Br J Cancer 92: 1915–1921. 15870709 10.1038/sj.bjc.6602594 PMC2361773

[22] Lombardi DP, Geradts J, Foley JF, Chiao C, Lamb PW, et al (1999) Loss of KAI1 expression in the progression of colorectal cancer. Cancer Res 59: 5724–5731. 10582691

[23] Kouzu Y, Uzawa K, Koike H, Saito K, Nakashima D, et al (2006) Overexpression of stathmin in oral squamous-cell carcinoma: correlation with tumour progression and poor prognosis. Br J Cancer 94: 717–723. 16495930 10.1038/sj.bjc.6602991 PMC2361217

[24] Verburg FA, Wäschle K, Reiners C, Giovanella L, Lentjes EG (2010) Heterophile antibodies rarely influence the measurement of thyroglobulin and thyroglobulin antibodies in differentiated thyroid cancer patients. Horm Metab Res 42: 736–739. 20486066 10.1055/s-0030-1254132

[25] Díaz-Rodríguez E, Álvarez-Fernández S, Chen X, Paiva B, López-Pérez R, et al (2011) Deficient spindle assembly checkpoint in multiple myeloma. PLoS One 6: e27583. 22132115 10.1371/journal.pone.0027583 PMC3223182

[26] Taylor WR, Stark GR (2001) Regulation of the G2/M transition by p53. Oncogene 20: 1803–1815. 11313928 10.1038/sj.onc.1204252

[27] Shames DS, Girard L, Gao B, Sato M, Lewis CM, et al (2006) A genome-wide screen for promoter methylation in lung cancer identifies novel methylation markers for multiple malignancies. PLoS Med 3: e486. 17194187 10.1371/journal.pmed.0030486 PMC1716188

[28] Collins I, Garrett MD (2005) Targeting the cell division cycle in cancer: CDK and cell cycle checkpoint kinase inhibitors. Curr Opin Pharmacol 5: 366–373. 15964238 10.1016/j.coph.2005.04.009

[29] Pines J, Hunter T (1989) Isolation of a human cyclin cDNA: evidence for cyclin mRNA and protein regulation in the cell cycle and for interaction with p34cdc2. Cell 58: 833–846. 2570636 10.1016/0092-8674(89)90936-7

[30] Yam CH, Fung TK, Poon RY (2002) Cyclin A in cell cycle control and cancer. Cell Mol Life Sci 59: 1317–1326. 12363035 10.1007/s00018-002-8510-y PMC11337442

[31] Shubbar E, Kovács A, Hajizadeh S, Parris TZ, Nemes S, et al (2013) Elevated cyclin B2 expression in invasive breast carcinoma is associated with unfavorable clinical outcome. BMC Cancer 13: 1. 23282137 10.1186/1471-2407-13-1 PMC3545739

[32] Pagano M, Pepperkok R, Verde F, Ansorge W, Draetta G (1992) Cyclin A is required at two points in the human cell cycle. EMBO J 11: 961–971. 1312467 10.1002/j.1460-2075.1992.tb05135.x PMC556537

[33] Sun M, Mo W, Fu X, Wu G, Huang Y, et al (2010) System biology analysis of cell cycle pathway involved in hepatocellular carcinoma. Front Biosci (Schol Ed) 2: 1127–1144. 20515845 10.2741/s122

[34] Parker LL, Piwnica-Worms H (1992) Inactivation of the p34cdc2-cyclin B complex by the human WEE1 tyrosine kinase. Science 257: 1955–1957. 1384126 10.1126/science.1384126

[35] Ruiz EJ, Vilar M, Nebreda AR (2010) A two-step inactivation mechanism of Myt1 ensures CDK1/cyclin B activation and meiosis I entry. Curr Biol 20: 717–723. 20362450 10.1016/j.cub.2010.02.050

[36] Nurse P (1990) Universal control mechanism regulating onset of M-phase. Nature 344: 503–508. 2138713 10.1038/344503a0

[37] Gautier J, Solomon MJ, Booher RN, Bazan JF, Kirschner MW (1991) cdc25 is a specific tyrosine phosphatase that directly activates p34cdc2. Cell 67: 197–211. 1913817 10.1016/0092-8674(91)90583-k

[38] Coleman TR, Dunphy WG (1994) Cdc2 regulatory factors. Curr Opin Cell Biol 6: 877–882. 7880537 10.1016/0955-0674(94)90060-4

