# Supplementary figures and images for: Overexpression of LIM and SH3 Protein 1 Leading to Accelerated G2/M Phase Transition Contributes to Enhanced Tumourigenesis in Oral Cancer
Source: PLoS One. 2013 Dec 26;8(12):e83187. doi: 10.1371/journal.pone.0083187 (PMC3873298; doi:10.1371/journal.pone.0083187)

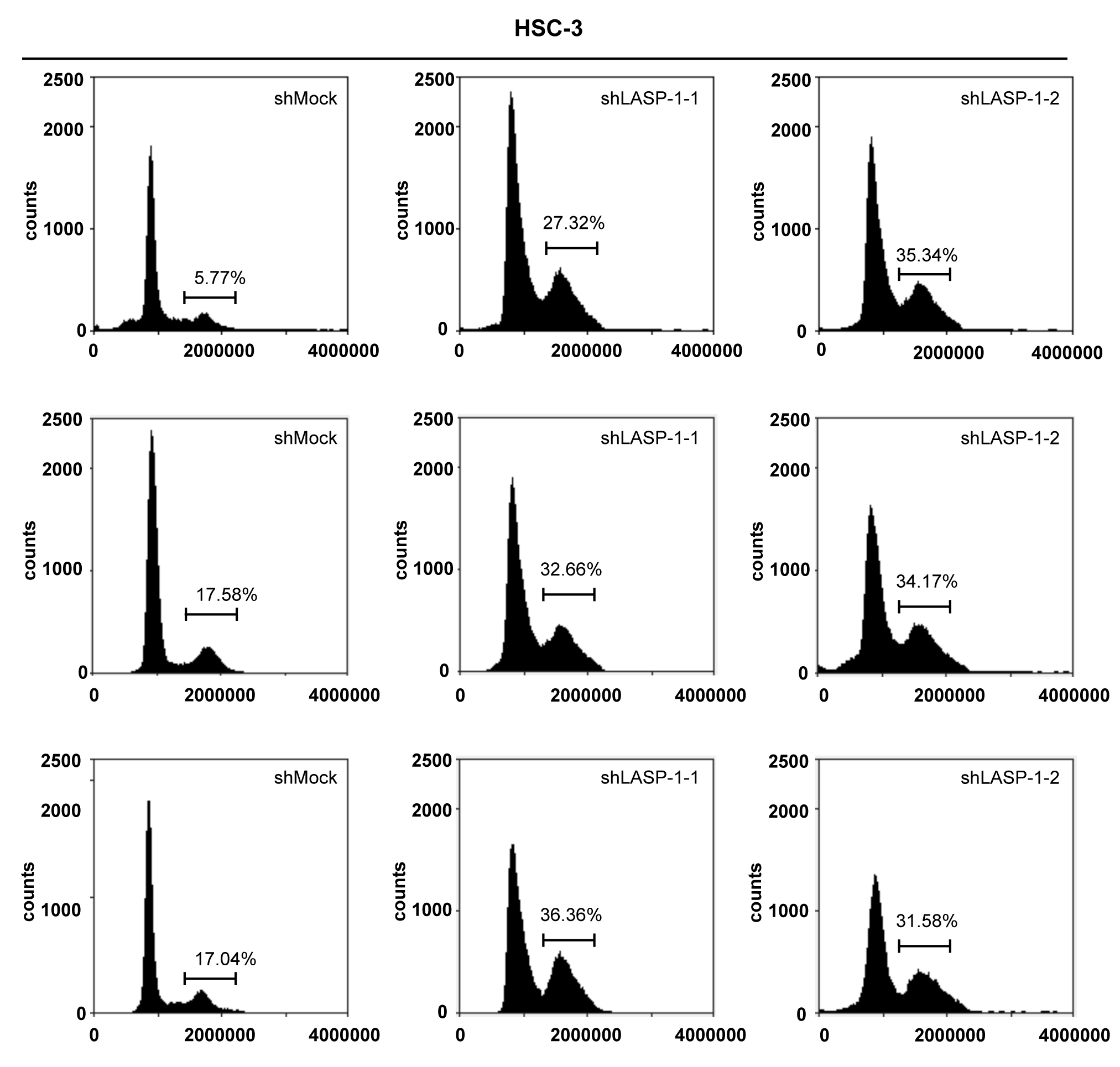

Supplement: Figure S1 — FACS analysis of shMock- and shLASP-1-transfected HSC-3 cells. The percentage of the G2/M phase in shLASP-1-transfected HSC-3 cells was higher than in shMock-transfected cells. (TIF) [file pone.0083187.s001.tif]

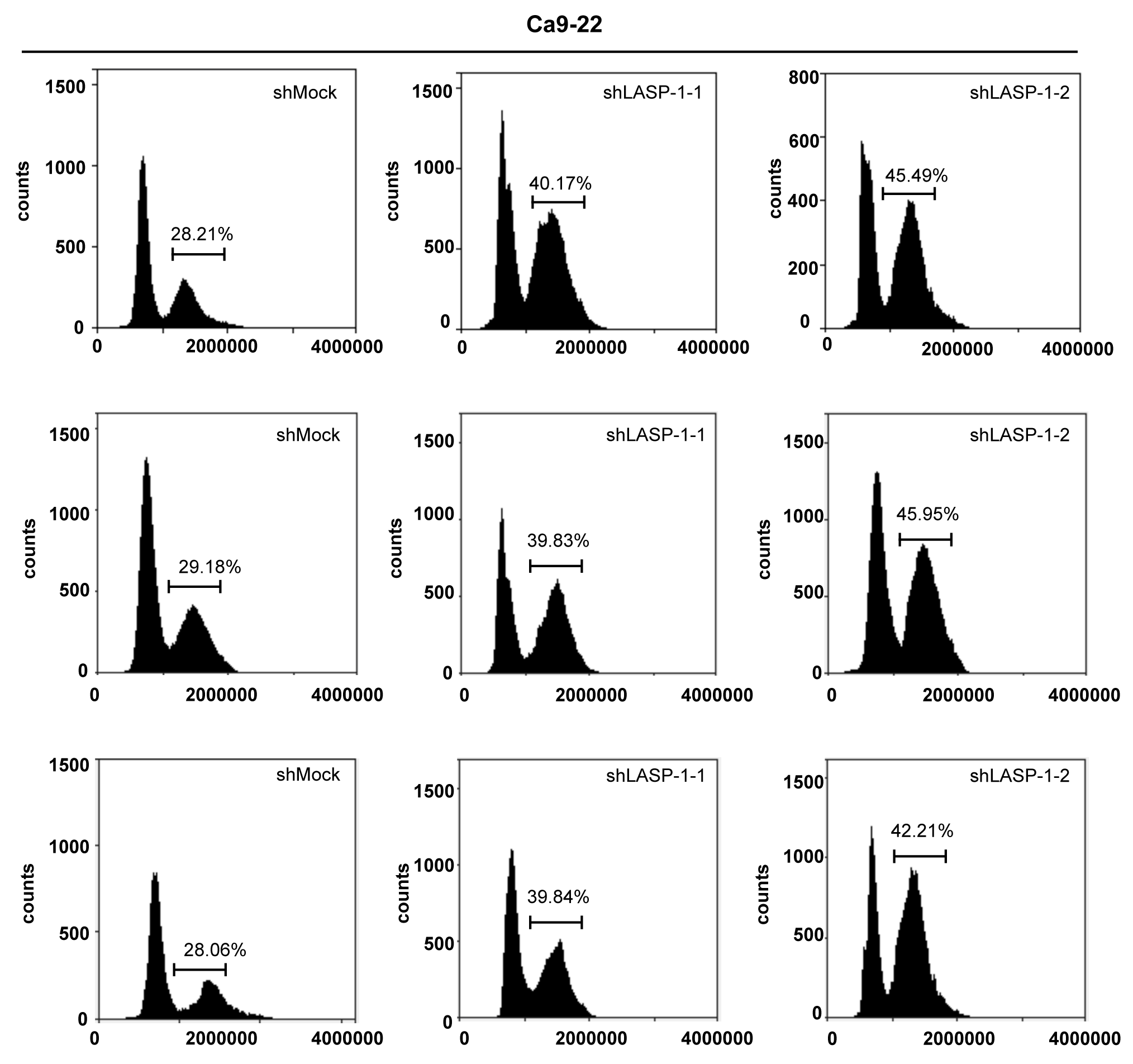

Supplement: Figure S2 — FACS analysis of shMock- and shLASP-1-transfected Ca9-22 cells. The percentage of the G2/M phase in shLASP-1-transfected Ca9-22 cells was higher than in shMock-transfected cells. (TIF) [file pone.0083187.s002.tif]
